# Bacterial Infections in Patients With Severe Alcohol‐Associated Hepatitis: Drivers of Organ Failure and Mortality

**DOI:** 10.1111/liv.70111

**Published:** 2025-05-07

**Authors:** Laura Buttler, Jan Stange, Nikolaos Pyrsopoulos, Tarek Hassanein, Heiner Wedemeyer, Benjamin Maasoumy, Markus Busch, the VTL‐308 study group

**Affiliations:** ^1^ Department of Gastroenterology, Hepatology, Infectious Diseases and Endocrinology Hannover Medical School Hannover Germany; ^2^ Center for Extracorporeal Organ Support (CEOS), Biomedical Research Center, Department of Nephrology University of Rostock Rostock Germany; ^3^ Liver Disease in New Jersey NYU Grossman School of Medicine, NYU Langone Transplant Institute New York USA; ^4^ Southern California Research Center Coronado California USA; ^5^ German Center for Infection Research (DZIF) Hannover‐Braunschweig Germany

**Keywords:** alcohol‐associated liver disease (ALD), corticosteroids in alcohol‐associated hepatitis, fungal infections in sAH, infections in alcohol‐associated hepatitis, predictive modelling in sAH, severe alcohol‐associated hepatitis (sAH)

## Abstract

**Background:**

Severe alcohol‐associated hepatitis (sAH) is a life‐threatening condition with limited treatment options. Although corticosteroids offer some benefit in short‐term survival, their use remains controversial due to concerns about increased infection risk. Infections are a major cause of mortality in sAH; however, the reasons for this remain unclear.

**Methods:**

A post hoc analysis of the prospective VTL‐308 multicentre trial on 151 patients with sAH was performed. Competing‐risk models evaluated predictors of infections, the influence of corticosteroids on infection risk, and the impact of infections on the clinical outcomes up to 1 year of follow‐up.

**Results:**

Among 151 patients, 90 (59.6%) developed infections. The most frequent were urinary tract (34.4%) and bloodstream (30%) infections. The causative pathogen was isolated in 40 patients, with *Enterococcus* spp. being the most common (35%). Fungal infections were detected in 19 (12.6%) patients. Corticosteroid use was not associated with increased bacterial (subdistribution‐hazard ratio [sHR] =0.74; 95% Confidence Interval (CI): 0.42–1.33; *p* = 0.32) or fungal infection risk (sHR = 1.74; 95% CI: 0.59–5.15; *p* = 0.31). Infections significantly increased multi‐organ failure (MOF) in the univariate (sHR = 2.31; 95% CI: 1.03–5.17; *p* = 0.04) and multivariate models (sHR = 2.46; 95% CI: 1.12–5.39; *p* = 0.03). 37.8% of infected patients died versus 13.1% of non‐infected patients. Bacterial infections strongly predicted mortality, with sHRs ranging from 5.22 to 7.78, indicating a five‐ to eight‐fold increased risk of death (*p* < 0.001).

**Conclusions:**

Infections in sAH are central drivers of MOF and mortality. Our findings highlight infections as an independent risk factor unaffected by corticosteroid use, addressing previous concerns about the safety of this treatment.

**Abbreviations**
ACLF: acute‐on‐chronic liver failure
AH: alcohol‐associated hepatitis
ALD: alcohol‐associated liver disease
ALT: alanine aminotransferase
AP: alkaline phosphatase
AST: aspartate aminotransferase
BSI: bloodstream infection
CI: confidence interval
ELAD: extracorporeal liver assist device
ESLD: end‐stage liver disease
GAHS: Glasgow alcoholic hepatitis score
HR: hazard ratio
INR: international normalised ratio
LTx: liver transplantation
MDF: Maddrey discriminant function
MELD: model for end‐stage liver disease
MOF: multi‐organ failure
sAH: severe alcohol‐associated hepatitis
SBP: spontaneous bacterial peritonitis
SOC: standard of care
spp.: species
UTI: urinary tract infection
WBC: white blood cells

**Summary**

- Bacterial infections are common and dangerous in patients with severe alcohol‐associated (sAH).
- In this study, we found that these infections strongly increase the risk of death and organ failure, more than any other factor.
- Early detection and treatment of infections could help save lives in this high‐risk group.

## 1 Background

Severe alcohol‐associated hepatitis (sAH) is a life‐threatening form of alcohol‐associated liver disease (ALD) [1], characterised by acute hepatic inflammation, systemic immune activation, and frequent progression to multi‐organ failure (MOF), with a 90‐day mortality rate as high as 50% [2].

Corticosteroids offer short‐term survival benefits [3], but their role is controversial due to concerns about infection risk. Bacterial infections are common in patients with sAH, with reported rates up to 50% [4], and are associated with poor outcomes. However, their incidence and clinical relevance—particularly in the context of corticosteroid therapy—remain incompletely understood. Prior studies have yielded conflicting results; some report increased infection rates and severity during or after corticosteroid use [5], while others found no significant association [6].

Given this uncertainty, we aimed to better characterise the burden and consequences of infections in sAH. Our objectives were to identify clinical predictors of infection, with a particular focus on corticosteroid use, and to examine whether infections independently contribute to organ failure and mortality in this vulnerable patient population.

## 2 Methods

### 2.1 Study Population

A post hoc analysis was performed on infectious episodes using data from the VTL‐308 trial, a multicentre, randomised, open‐label study conducted between 2016 and 2018 [7]. The 44 participating study centres are displayed in Figure S1. In this trial, 151 patients with sAH were enrolled. Patients were diagnosed with sAH in a two‐step process: first, based on recent alcohol use (≤ 6 weeks before admission) consistent with alcohol‐induced liver decompensation (AILD); second, by fulfilling either histological confirmation or ≥ 2 typical clinical features (hepatomegaly, AST>ALT, ascites, leukocytosis), alongside a bilirubin ≥ 16 mg/dL and a Maddrey discriminant function (DF) score ≥ 32. The study participants were randomised at a 1:1 ratio to receive either standard of care (SOC) treatment plus Extracorporeal Liver Assist Device (ELAD) therapy (ELAD group) or SOC treatment alone (control group). The ELAD system represents a hepatic cell‐based liver treatment and consists of four cartridges, each containing 110 g of cloned, immortalised human hepatoblastoma cells of the HepG2 line (VTL C3A cells). Blood was drawn by the system via a dual‐lumen central venous catheter, and the blood cells were separated from the ultrafiltrate. The latter circulated through the cartridges before both the ultrafiltrate and cellular blood components returned to the respective patient via the dual‐lumen catheter. However, the treatment did not have any impact on the clinical course. The VTL‐308 study protocol specified that corticosteroids were administered according to the Lille procedure, with continuation decided after 7 days based on bilirubin response. Steroid use was left to the discretion of the treating investigators. The presence of infection was not a formal exclusion criterion for initiating corticosteroids. While prophylactic antibiotic use was not part of the protocol, antibiotics were administered at the investigator's discretion in cases of confirmed infections. Details on the timing of antibiotic treatment were not systematically captured in the VTL‐308 trial. The detailed inclusion and exclusion criteria are presented in Table S1.

### 2.2 Definition of Infections

Infections were recorded as serious adverse events (SAEs) in the VTL‐308 trial when at least two of the following three criteria were met: [1] clinical suspicion of infection (e.g., new‐onset fever, organ‐specific manifestations), [2] laboratory abnormalities compatible with infection (e.g., leukocytosis, rising CRP), and [3] microbiological confirmation (e.g., positive cultures from blood, urine, or other sterile compartments). Diagnostic decisions were made by the local investigators.

### 2.3 Study Outcomes and Statistical Approaches

This study investigated three primary outcomes: (i) predictors of infections, (ii) infection‐related mortality, and (iii) infection‐associated MOF. The statistical approach for each outcome was defined as follows:

- Predictors of infections: To identify factors independently associated with infections, a competing risk regression model was constructed, treating Liver transplantation (LTx) and death as competing events. To particularly evaluate the impact of corticosteroid treatment on the risk for bacterial and fungal infections, patients were divided into steroid and no steroid groups depending on their respective baseline medication. Due to the fact that steroid therapy usually does not last for the entire observation period, the analysis was repeated by a time‐censored corticosteroid use, where patients were censored upon corticosteroid initiation or discontinuation during follow‐up. This allowed us to account for corticosteroid timing and more accurately assess its potential role in increasing the risk of infection. The competing risk analyses were performed univariate and multivariate. For the multivariate analysis of infection predictors, variables were selected based on clinical plausibility, prior literature, and data availability. As no significant univariate associations were observed, corticosteroid use, baseline MELD score, and recent infection history were included in an exploratory multivariate model. Given that 44 patients had infections at baseline, they were excluded from this analysis to evaluate only new infections. The results were reported as subdistribution‐hazard ratios (sHRs) with 95% confidence intervals (CIs).
- Infection‐related mortality: To assess the clinical relevance of infections in sAH, their association with mortality was investigated. Two statistical approaches were used: First, a competing risk model was used, treating LTx as a competing event. A second approach entailed the implementation of a Cox proportional hazard regression with time‐dependent covariates. This method was utilised to verify the accuracy of the analysis, whilst simultaneously addressing the issue of immortal time bias by assigning patients to the infection group only after infection onset. For the multivariate models examining infection‐related mortality, infection was included as the primary variable of interest. The other covariates (age, bilirubin, INR, urea, WBC and liver cirrhosis) were included based on their clinical relevance and prior evidence [6, 7, 8]. Results were reported as sHRs (competing risk model) and HRs (Cox regression) with 95% CIs.
- Infection‐associated MOF: MOF was defined as the simultaneous presence of three or more organ failures according to the Chronic Liver Failure Consortium Organ Failure (CLIF‐C OF) criteria. The presence of renal and coagulation failure, hemodynamic instability, and mechanical ventilation were defined as exclusion criteria according to the VTL‐308 study protocol; therefore, none of the patients presented with MOF at baseline. Analogous to the analysis of infection‐related mortality, two statistical approaches were utilised: A competing risk model with the competitors LTx and death as well as a Cox proportional hazard regression with time‐dependent covariates to exclude the immortal time bias. Multivariate analyses controlled for age, bilirubin, International Normalised Ratio (INR), leukocytes, urea, and the presence of liver cirrhosis, all selected for their established relevance in predicting mortality in patients with sAH [6, 7, 8]. Results were reported as sHRs (competing risk model) and HRs (Cox regression) with 95% CIs.

All patients were followed up for 90 days. After discharge, ongoing prospective data collection was ensured by weekly home visits or inpatient monitoring in case of rehospitalisation. To minimise the risk of missing events, subjects were censored at the time point of loss to follow‐up in all analyses. To ensure data validity, subgroup analyses for the ELAD and standard of care group were added to the Supporting Information (Table S2A–D, Figure S2).

### 2.4 Ethics Statement

The VTL‐308 trial (NCT02612428) was approved by the local ethics committees of each participating centre and was conducted in accordance with the Declarations of Helsinki and Istanbul. Written informed consent was obtained from all participants prior to their inclusion in the study.

### 2.5 Statistics

All statistical analyses were performed using R Statistical Software (version 4.2.0, R Foundation for Statistical Computing, Vienna, Austria) with the ‘tableone’ package for descriptive statistics, IBM SPSS Statistics (Version 28, IBM, New York) for data visualisation, and Rcmdr with the EZR plugin for regression and competing risk analyses. GraphPad Prism (version 10.0) was used to generate bar charts.

Descriptive statistics were used to summarise baseline patient characteristics. Continuous variables were reported as medians with interquartile ranges (IQRs) and compared using the Mann–Whitney *U* test due to non‐normal distribution. Categorical variables were reported as counts and percentages and compared using the chi‐square or Fisher's exact test, as appropriate.

## 3 Results

### 3.1 Cohort Characterisation

In total, 151 patients with sAH were enrolled in this study. The patients had a median baseline MELD of 25, a median Maddrey Discriminant Function (MDF) of 63, and a median age of 40 years. Median follow‐up time was 90 days. Overall, a number of 35 (23.2%) patients fulfilled the criteria for MOF during 1 year of follow‐up, 4 (2.6%) underwent LTx, and 42 (27.8%) patients died. Baseline clinical and biological characteristics, including organ dysfunction and relevant scoring systems, are summarised in Table 1.

**TABLE 1:** Baseline characteristics of the 151 patients analysed in this study, comparing those with corticosteroid treatment and those without steroid therapy.

	All patients (*n* = 151)	No steroids (*n* = 88)	Steroids (*n* = 63)	*p* ^a^
Age	40.00 (34.00, 45.00)	42.00 (35.00, 46.00)	39.00 (33.50, 44.50)	0.100
Male sex	91 (60.3)	52 (59.1)	39 (61.9)	0.857
Maddrey Score	63.00 (50.10, 79.40)	65.05 (51.88, 81.75)	62.50 (50.35, 76.45)	0.493
GAHS	9.00 (8.00, 9.00)	9.00 (8.00, 9.00)	8.00 (8.00, 9.00)	0.109
MELD	25 (23, 27)	25.00 (23.00, 27.25)	25.00 (23.00, 27.00)	0.412
WBC (10^9^/L)	12.20 (8.10, 16.90)	11.80 (8.09, 16.74)	12.88 (8.30, 17.44)	0.446
Haemoglobin (g/dL)	10.20 (8.80, 11.50)	9.90 (8.60, 11.22)	10.60 (9.10, 11.65)	0.069
Platelets (10^9^/L)	156.00 (98.00, 212.00)	159.00 (94.75, 227.00)	154.00 (105.00, 194.00)	0.944
Prothrombin time (sec)	20.50 (17.90, 23.70)	20.30 (18.00, 23.95)	20.80 (17.70, 22.70)	0.564
INR	1.80 (1.50, 2.10)	1.80 (1.54, 2.20)	1.80 (1.50, 2.00)	0.445
Sodium (mEq/L)	135.00 (131.00, 138.00)	135.00 (131.00, 138.00)	135.00 (132.00, 137.00)	0.943
Creatinine (mg/dL)	0.69 (0.53, 0.90)	0.70 (0.60, 0.90)	0.64 (0.52, 0.88)	0.237
Bilirubin (mg/dL)	24.12 (19.62, 28.30)	22.00 (19.08, 28.87)	24.92 (20.97, 27.97)	0.364
Albumin (g/dL)	2.70 (2.40, 3.20)	2.70 (2.30, 3.20)	2.70 (2.40, 3.20)	0.896
AST (U/L)	124.00 (89.00, 168.50)	121.00 (95.50, 159.50)	135.00 (84.50, 176.00)	0.724
ALT (U/L)	43.00 (30.00, 67.00)	42.00 (29.00, 61.50)	45.00 (31.50, 69.50)	0.303
De ritis ratio	2.86 (1.97, 3.64)	2.85 (2.04, 4.04)	2.86 (1.84, 3.43)	0.597
AP (U/L)	173.00 (120.50, 221.50)	153.50 (112.50, 206.50)	194.00 (132.75, 240.25)	0.008
Urea (mg/dL)	21.40 (14.69, 33.17)	21.40 (14.69, 34.24)	21.40 (14.98, 29.52)	0.758
Alpha fetoprotein (ng/mL)	4.00 (3.00, 5.40)	3.80 (3.00, 4.99)	5.00 (3.39, 6.66)	0.044
Liver length (cm)	20.20 (17.85, 23.65)	20.00 (17.00, 22.25)	20.80 (18.15, 24.50)	0.050
Liver cirrhosis	106 (70.2)	60 (68.2)	46 (73.0)	0.646
Pentoxifylline within 7 days before baseline	25 (16.6)	15 (17.0)	12 (19.0)	0.919
ELAD treatment	56 (50.3)	49 (55.7)	27 (42.9)	0.165
Bacterial infection at baseline	44 (29.1)	25 (28.4)	19 (30.2)	0.959
Ascites within 7 days before baseline	131 (86.8)	73 (83.0)	58 (92.1)	0.166
Moderate/severe ascites within 7 days before baseline	57 (37.7)	34 (38.6)	23 (36.5)	0.924
Hepatic encephalopathy within 7 days before baseline	69 (45.7)	41 (46.6)	28 (44.4)	0.924
Last alcohol intake before admission (weeks)	1.00 (1.00, 3.00)	1.00 (1.00, 3.00)	1.00 (1.00, 2.00)	0.270
Alcohol intake during follow‐up	21 (13.9)	12 (13.6)	9 (14.3)	1.000

*Note:* Values as number (percentage) or median (IQR).

Abbreviations: ALT, alanine aminotransferase; AP, alkaline phosphatase; AST, aspartate aminotransferase; ELAD, extracorporeal liver assist device; GAHS, Glasgow alcoholic hepatitis score; INR, international normalised ratio; MELD, model for end‐stage liver disease; WBC, white blood cells.

^a^ Chi‐squared test was used for comparison of categorical variables, Mann–Whitney *U* for continuous parameters.

### 3.2 Incidence and Types of Infections

**FIGURE 1:**
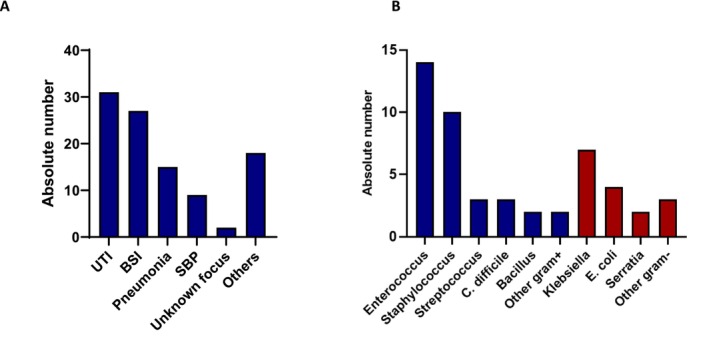
Types of infections in the infection group (*n* = 90) (A). Frequency of different pathogens identified in infected patients (grampositive and gramnegative) (B). BSI, bloodstream infection; HR, hazard ratio; SBP, spontaneous bacterial peritonitisUTI, urinary tract infection.

Ninety out of 151 patients (59.6%) developed an infection within the first 90 days. Most infections occurred during hospitalisation (*n* = 60; 66.7%), whereas fewer infections were already present at admission (*n* = 17; 18.9%) or were acquired after discharge (*n* = 13; 14.4%). The most common infections were urinary tract infections (UTI), occurring in 31 patients (34.4%), followed by bloodstream infections (BSI) in 27 patients (30%), and pneumonia in 15 patients (16.7%). Peritonitis occurred in nine patients (10%), and infections with an unidentified focus were noted in two patients (2.2%). Additionally, other infections were reported in 18 patients (20%). The infection types are listed in Table 2 and Figure 1A. Furthermore, fungal infections occurred in 19 patients (12.6%), with oral candidiasis being most common (*n* = 8; 42.1%), followed by fungal skin infections (*n* = 4; 21.1%) and oesophageal candidiasis (*n* = 3; 15.8%).

**TABLE 2:** Distribution of various types of infections in the infection group (*n* = 90).

	Infection group (*n* = 90)
Urinary tract infection	31 (34.4)
Bloodstream infection	27 (30.0)
Pneumonia	15 (16.7)
Peritonitis	9 (10.0)
Unknown focus	2 (2.2)
Others	18 (20.0)

*Note:* Values as number (percentage). Results in more than 100% due to presence of coinfections.

### 3.3 Microbial Pathogens

Positive microbiological cultures were reported in 40 out of the 90 infected patients (44.44%). Co‐infections occurred frequently, resulting in a total exceeding 100%. The causative pathogens were identified in 40 patients. Among gram‐positive bacteria, *Enterococcus* species (spp.) were the most common (35%), with vancomycin‐resistant *Enterococcus* (VRE) isolated in three patients. *Enterococcus* infections were followed by other *Staphylococcus* spp. (17.5%), *Staphylococcus aureus* (7.5%), which was methicillin‐resistant (MRSA) in one case, *Streptococcus* spp. (7.5%), *Clostridium difficile* (*C. diff*) (7.5%), and *Bacillus* (5%). Other gram‐positive bacteria accounted for 5% of cases. Among gram‐negative bacteria, *Klebsiella* was identified in 17.5% of cases, *E. coli* in 10%, *Serratia* in 5%, and other gram‐negative bacteria present in 7.5% of infections. The isolated pathogens are summarised in Table 3 and Figure 1B.

**TABLE 3:** Pathogens identified in patients with infections (gram‐positive and gram‐negative).

	Identified pathogens (detected in 40 patients with infection)
Gram+	
*Enterococcus* sp.	14 (35.0)
*Staphylococcus aureus*	3 (7.5)
*Staphylococcus* (others than *S. aureus* )	7 (17.5)
*Streptococcus* sp.	3 (7.5)
*Clostridoides difficile*	3 (7.5)
*Bacillus*	2 (5.0)
Other Gram+	2 (5.0)
Gram−	
*Klebsiella*	7 (17.5)
*Escherischia coli*	4 (10.0)
*Serratia*	2 (5.0)
Other Gram−	3 (7.5)

*Note:* Values as number (percentage).

### 3.4 Risk Factors for Infections

Corticosteroid use, MELD, or prior infections were not significantly associated with an elevated infection risk in the univariate and multivariate models during the 90‐day follow‐up period. This was consistent even when adjusting for the timing of corticosteroid administration, where patients were censored at the point of steroid initiation or discontinuation. Details of the variables, along with their respective hazard ratios, confidence intervals, and *p*‐values, are shown in Table S3.

**FIGURE 2:**
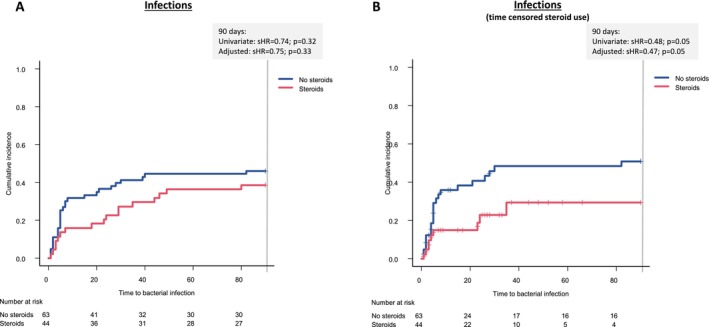
Incidences of infections were not increased in patients receiving corticosteroid therapy. The figures illustrate the results of competing risk analyses. In a first approach, patients were divided into steroid or no steroid groups depending on their baseline medication (A). In a second model, the patients were also assigned to groups depending on their respective baseline medication, but were censored with the time point of steroid discontinuation or with the new onset of steroids within the observation period (B). HR, hazard ratio.

**FIGURE 3:**
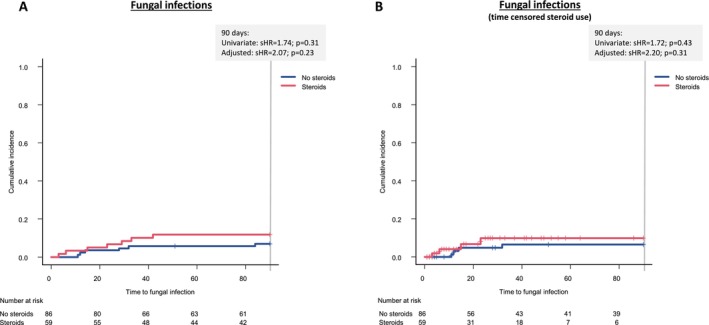
Corticosteroid was not linked to higher incidences of fungal infections. Steroid and no steroid groups were defined based on the respective baseline medication (A) and patients were censored if steroid therapy had been started/stopped during follow‐up (B).

Corticosteroids were administered to a number of 88 patients at baseline, with a median treatment duration of 18 days and a median cumulative dose of 595 mg. Dose and duration of baseline corticosteroid treatment in patients with and without infections are summarised in Table 4. Both steroid dose and duration were comparable between groups, with *p* values of 0.38 and 0.31, respectively. Notably, corticosteroid therapy was not linked to an increased risk of bacterial infections (sHR = 0.74; 95% CI: 0.42–1.33; *p* = 0.32) or to a higher likelihood of fungal infections (sHR = 1.74; 95% CI: 0.59–5.15; *p* = 0.31) within 90 days (Figures 2, 3). This observation was confirmed in the multivariate competing risk model adjusted for MELD and previous infections (bacterial infections: sHR = 0.75; 95% CI: 0.42–1.34; *p* = 0.33; fungal infections: HR = 2.07; 95% CI: 0.63–6.73; *p* = 0.23) (Table 5A,B).

**TABLE 4:** Dose and duration of baseline corticosteroid treatment comparing patients with and without infection.

	All patients (*n* = 63 with steroid treatment)	Infection group (*n* = 36 with steroid treatment)	No infection group (*n* = 27 with steroid treatment)	*p*
Median cumulative dose of steroid treatment (mg)	595.00 (240.00–1163.50)	40.00 (14.25–156.25)	78.00 (24.00–134.50)	0.382
Median cumulative dose of prednisolone (mg)	685.00 (240.00–1167.00)	40.00 (14.75–152.75)	78.00 (24.00–134.50)	0.609
Median cumulative dose of methylprednisolone (mg)	500.00 (296.00–950.00)	52.00 (2.00–56)	36.00 (25.00–168.00)	0.881
Median duration of steroid treatment (days)	18.00 (6.00–38.50)	10.00 (5.00–34.25)	16.00 (7.50–30.50)	0.311
Median duration of prednisolone treatment (days)	20.00 (6.00–39.00)	10.00 (4.75–36.00)	23.00 (6.00–33.50)	0.420
Median duration of methylprednisolone treatment (days)	12.50 (8.25–14.75)	13.00 (5.00–14)	9.00 (8.50–13.50)	0.881

*Note:* Values as median (IQR). Mann–Whitney *U* test was used for group comparisons.

**TABLE 5:** Corticosteroid therapy was neither associated with an increased risk for bacterial (A) nor for fungal infections (B).

	Univariate				Multivariable			
	sHR	Lower 95% CI	Upper 95% CI	*p*	HR	Lower 95% CI	Upper 95% CI	*p*
(A)								
90 days								
Steroids	0.74	0.42	1.33	0.320	0.75	0.42	1.34	0.330
MELD	1.03	0.90	1.17	0.690	1.03	0.90	1.17	0.710
Previous infection (30 days)	0.88	0.41	1.86	0.730	0.89	0.42	1.90	0.770
Liver cirrhosis	0.93	0.51	1.71	0.820	0.91	0.49	1.67	0.760

	HR	Lower 95% CI	Upper 95% CI	*p*	HR	Lower 95% CI	Upper 95% CI	*p*
Time‐censored corticosteroid use—90 days								
Steroids	0.48	0.22	1.01	0.054	0.47	0.22	1.01	0.054
MELD	0.99	0.86	1.16	0.940	1.00	0.86	1.16	0.970
Previous infection (30 days)	0.79	0.36	1.74	0.560	0.79	0.35	1.79	0.580
Liver cirrhosis	0.85	0.42	1.73	0.660	0.80	0.39	1.68	0.560

	Univariate				Multivariable			
	sHR	Lower 95% CI	Upper 95% CI	*p*	HR	Lower 95% CI	Upper 95% CI	*p*
(B)								
90 days								
Steroids	1.74	0.59	5.15	0.310	2.07	0.63	6.73	0.230
MELD	1.03	0.84	1.26	0.780	1.13	0.90	1.41	0.300
Previous infection (30 days)	< 0.001	< 0.001	< 0.001	0.000	< 0.001	< 0.001	< 0.001	< 0.001
Liver cirrhosis	0.33	0.11	0.97	0.043	0.27	0.08	0.85	0.026

	HR	Lower 95% CI	Upper 95% CI	*p*	HR	Lower 95% CI	Upper 95% CI	*p*
Time‐censored corticosteroid use—90 days								
Steroids	1.72	0.45	6.57	0.430	2.20	0.48	10.17	0.310
MELD	1.11	0.83	1.49	0.470	1.26	0.88	1.80	0.210
Previous infection (30 days)	< 0.001	< 0.001	< 0.001	0.000	< 0.001	< 0.001	< 0.001	< 0.001
Liver cirrhosis	0.36	0.09	1.41	0.140	0.24	0.05	1.13	0.071

*Note:* The tables summarise hazard ratios (HR) and 95% confidence intervals (CI) from competing risk analyses for 90 days.

Abbreviation: MELD, model for end‐stage liver disease.

### 3.5 Impact of Infections on Mortality

**FIGURE 4:**
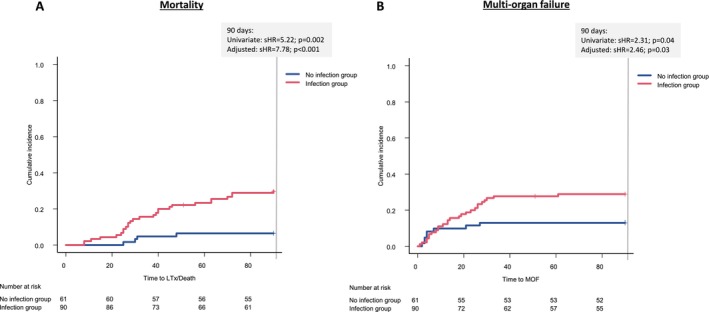
Infections were strongly linked to a drastic increase in mortality (A) and multi‐organ failure (B). HR, hazard ratio; LTx, liver transplantation; MOF, multi‐organ failure.

Competing risk analysis demonstrated a significant association between infections and increased mortality (Figure 4A). In the univariate analysis, infections were associated with a sHR of 5.22 for 90‐day mortality (95% CI: 1.83–14.90; *p* = 0.002), indicating a significant impact. This strong association persisted in the multivariate model (sHR = 7.78; 95% CI: 2.44–24.86; *p* < 0.001) (Table S4A). Additional covariates such as age, bilirubin, INR, and urea levels also significantly increased mortality risk. Among these, INR demonstrated a particularly high impact (sHR = 3.65; 95% CI: 1.19–11.14; *p* = 0.02), whereas the leukocyte count did not show a statistically significant effect (sHR = 0.98; 95% CI: 0.95–1.02; *p* = 0.39).

Cox regression analysis with time‐dependent covariates confirmed the significant effect of infection on mortality. In the 90‐day analysis, infections showed an HR of 4.8 in the univariate model (95% CI: 1.99–11.57; *p* < 0.001), which increased to 7.19 in the multivariate model (95% CI: 2.83–18.25; *p* < 0.001). The INR had a notable HR of 3.52 (95% CI: 1.14–10.83; *p* = 0.03), whereas urea remained a consistent predictor (HR = 1.03; 95% CI: 1.02–1.05; *p* < 0.001) (Table S4A).

Regarding fungal infections, no impact on mortality was observed both in the univariate (sHR = 0.94; 95% CI: 0.36–2.49; *p* = 0.9) and multivariate (sHR = 0.63; 95% CI: 0.14–2.87; *p* = 0.55) competing risk models (Figure S3A). This was in line with the results of the univariate (HR = 1.46; 95% CI: 0.56–3.77; *p* = 0.44) and multivariate (HR = 1.36; 95% CI: 0.49–3.76; *p* = 0.55) Cox regression analyses (Table S4A).

### 3.6 Impact of Infections on Organ Function

In the overall 35 (23.2%) patients with MOF, the most frequent types of contributing organ failure were hepatic (*n* = 34; 97.1%), followed by coagulation (*n* = 19; 54.3%) and circulatory failure (*n* = 18; 51.4). Respiratory, renal, and cerebral failure were observed in a number of 17 (48.6%), 16 (45.7%) and 13 (37.1%) subjects, respectively. The competing risk model revealed that infections were associated with a sHR of 2.31 (95% CI: 1.03–5.17; *p* = 0.04) for MOF in the univariate analysis, which persisted in the multivariate model (sHR = 2.46; 95% CI: 1.12–5.39; *p* = 0.03) after adjusting for age, bilirubin, INR, leukocytes, urea, and history of liver cirrhosis. (Figure 4B).

Cox regression confirmed that infections significantly increased the risk of MOF, with an HR of 1.99 (95% CI: 1.00–3.96; *p* = 0.05) in the univariate model and 2.46 (95% CI: 1.18–5.11; *p* = 0.02) in the multivariate model. Consistent with the high frequency of renal failure, urea levels were significantly associated with MOF risk (Competing risk: sHR = 1.02; 95% CI: 1.00–1.04; *p* = 0.02; Cox regression: HR = 1.02; 95% CI: 1.00–1.04; *p* = 0.03). Hepatic dysfunction, reflected by bilirubin level, was consistently linked to MOF risk (Competing risk: sHR = 1.05; 95% CI: 1.01–1.10; *p* = 0.02; Cox regression: HR = 1.06; 95% CI: 1.01–1.12; *p* = 0.02) (Table S5A).

In contrast, fungal infections were not associated with an elevated incidence of MOF in the univariate (sHR = 0.67; 95% CI: 0.20–2.28; *p* = 0.52) and multivariate competing risk models (sHR = 0.57; 95% CI: 0.17–1.88; *p* = 0.36) (Figure S3B) or in the univariate (HR = 0.43; 95% CI: 0.06–3.17; *p* = 0.41) and multivariate (HR = 0.44; 95% CI: 0.06–3.34; *p* = 0.43) Cox regression analyses (Table S5B), respectively.

## 4 Discussion

In this post hoc analysis of 151 patients from the VTL‐308 trial, we investigated the incidence, risk factors, and outcomes of infection in patients with sAH. Our findings emphasise the critical role of bacterial infections in sAH, challenging previous assumptions regarding the safety of corticosteroids concerning the risk of infection. Infections have emerged as a primary determinant of mortality, surpassing even traditional scoring systems such as the Glasgow Alcoholic Hepatitis Score (GAHS) in terms of predictive power. The high mortality rate is probably due to an increase in MOF caused by infection.

Nearly 60% of patients developed infections during 90‐day follow‐up, making it even more frequent than previously reported [4, 8]. In our study, *Enterococcus* spp. was the most common gram‐positive pathogen (35%). Particularly, *Enterococcus faecalis* has gained recognition as a relevant pathogen in liver disease and alcohol use disorders, which are often associated with antibiotic resistance. Under conditions of dysbiosis and immune deficiency, this commensal bacterium can transform into an opportunistic pathogen and cause relevant complications [9]. These could be further exacerbated by the emergence of multidrug‐resistant pathogens, which were also detected in some cases in our study.

The second most common gram‐positive pathogen was *Staphylococcus* spp. (17.5% other species, 7.5% *S. aureus* ). Although part of the skin flora, it is known to pose a threat to immunocompromised hosts. The presence of *Klebsiella* (17.5%) and *E. coli* (10%) as the most common gram‐negative bacteria aligns with previous research that identifies these pathogens as prevalent in end‐stage liver disease (ESLD), especially in hospital settings [10]. These infections can lead to hepatic decompensation and worsen clinical outcomes [11].

In our cohort, UTI was the most common infection type, likely attributable to immune compromise and frequent catheterization in hospitalised patients with sAH [12]. BSI, affecting 30% of our patients, was the second most common, consistent with the recognised high prevalence in patients with ESLD. In previous studies, BSI affected 4%–21% of patients [13], making it 10 times more common in cirrhotic patients than in non‐cirrhotic patients [14]. Increased gut permeability and immune dysfunction may make this population prone to developing BSI via an endogenous route. BSIs in cirrhotic patients are linked to high mortality, reaching up to 30% within 30 days of admission, with gram‐negative bacteria, particularly *E. coli* and *Klebsiella*, as leading culprits [15, 16]. The observed high incidence of infections in sAH patients, especially UTIs and BSI, is consistent with prior literature, which links impaired immune function in liver disease to infection vulnerability [17, 18].

Our results showed no significant increase in infection rates associated with corticosteroid therapy, including fungal infections, when used according to the Lille procedure [5]. This finding supports the evidence that corticosteroids do not increase infection‐related mortality, consistent with a previous meta‐analysis of smaller randomised trials [6]. However, this conclusion remains a subject of ongoing debate, as other studies have suggested a possible association between corticosteroid use and infections [3], emphasising the need for further investigation. Our study suggests that steroids do not predispose patients to infections. Instead, the immune dysfunction inherent to liver failure, potentially exacerbated by toxins such as bile acids, likely contributes to this susceptibility [19, 20, 21]. The lack of association between corticosteroid use and higher infection rates in our study may be explained by the immunological context of sAH. In acute hyperinflammatory states, corticosteroids can help rebalance the immune system by modulating cytokine activity rather than causing broad immunosuppression [22]. Since excessive cytokine‐driven activation itself contributes to immune dysfunction and infection risk, dampening this response may, paradoxically, stabilise host defense.

The other variables analysed in our study, which have been associated with infections in other clinical settings, did not show the same association with sAH. Elevated leukocyte levels do not predict infections in this population, possibly because leukocytosis is a common feature of sAH itself [23], reducing the specificity of this traditional marker for infection. An increased INR is generally associated with liver dysfunction [24, 25], which has been linked to infection [18]. Nevertheless, in our study, the INR did not show a significant association with infection risk. Although MELD is often correlated with poor outcomes in liver disease, including infection risk due to advanced liver failure [26], it was not significant in our competing risk model. Low albumin levels are a strong predictor of liver decompensation [27] and are generally associated with an increased risk of infection because hypoalbuminemia indicates poor nutritional status and immune function [28]. However, this did not appear to be significant in our study, which might be due to the moderately reduced albumin levels and the complexity of sAH with multiple interacting risk factors.

Infections in sAH are not mere complications but stand out as independent and dominant predictors of mortality, surpassing even traditional scoring systems such as the GAHS. Bacterial infections increase the risk of death 5‐ to 8‐fold, emphasising the importance of understanding the mechanisms underlying these poor outcomes. A key hypothesis is the cascading progression of organ failure with infections as the initial trigger. In our analysis, infections doubled to tripled the risk of MOF (2‐ to 2.5‐fold), which was significantly shorter than the observed mortality increase (5‐ to 8‐fold). This disparity likely reflects limitations in the strict CLIF‐C SOFA definition of MOF as dysfunction in three or more organ systems, which excludes patients succumbing to single‐organ failure or those without comprehensive organ assessments shortly before death, which is often the case in terminal events occurring post‐discharge. Infections disproportionately impact the renal, hepatic, and coagulation systems, which are the key components of MELD. Acute kidney injury (AKI), which may be better reflected by urea than creatinine in patients with acute‐on‐chronic liver failure (ACLF) [29], is a known driver of mortality in sAH [30]. Rising bilirubin levels signalled worsening hepatic dysfunction, frequently contributing to further organ failure [31], and an elevated INR, indicating liver and coagulation failure, was strongly predictive of both MOF and mortality. Infections likely set off a cascade in which failure in one organ system propagates dysfunction in another, amplifying the mortality risk [32]. This cascade hypothesis explains the disproportionately high mortality associated with bacterial infections in patients with sAH.

The ELAD system has previously been associated with modulation of systemic inflammation, in part by dampening the pro‐inflammatory environment through the secretion of interleukin‐1 receptor antagonist (IL‐1RA) [33]. However, we observed no significant differences between ELAD and SOC patients in terms of infection incidence, infection‐related mortality, or the impact of infections on organ failure (Table S2A–D, Figure S2), suggesting that ELAD therapy did not substantially alter the systemic response to infections in our study.

Our findings demonstrate that infections are the leading cause of clinical deterioration and death in this population. Given the difficulty in distinguishing sterile inflammation from true infection, we believe that the greater clinical risk lies in delayed recognition and undertreatment rather than overtreatment. This underscores the importance of early diagnostic vigilance and a low threshold for initiating antibiotic therapy, particularly in the presence of abrupt clinical or laboratory changes. On the other hand, prophylactic antibiotic strategies have not yet been evaluated in prospective studies, and routine use may contribute to antimicrobial resistance. Further studies are needed to determine whether these high‐risk patients may benefit from preemptive or prophylactic antibiotic treatment approaches.

The challenge of distinguishing true infection from sterile systemic inflammation, which constitutes a hallmark of the disease itself, furthermore reflects a major limitation inherent to studies in sAH. For example, the WBC count in our cohort was similar between ‘infection’ and ‘non‐infection’ groups. While only a subset of infections (40 out of 90) was microbiologically confirmed, infection reporting followed predefined criteria requiring a combination of clinical, laboratory, and, when available, microbiological evidence. Established diagnostic standards were applied for typical infection sites, including urinary tract infections, bloodstream infections, pneumonia, and spontaneous bacterial peritonitis. Nevertheless, the potential for misclassification remains and must be acknowledged when interpreting infection‐related endpoints in sAH. Furthermore, our study is limited by its reliance on data from a single multicentre randomised controlled trial. While post hoc analyses inherently limit causal inferences, our use of prospectively collected data and competing risk models strengthens the validity of our findings. The sample size represents one of the largest available cohorts of sAH patients with detailed infection data. However, the relatively small sample size reveals a limitation of our study. Therefore, it cannot be discounted that the sample size might have been too low to attain statistical significance for some minor effects. Of note, strong statistically significant associations between infections and clinical outcomes were detected, indicating sufficient power for the main and clinically relevant endpoints of our study. Although the number of fungal infections was small, precluding definitive conclusions, our study underscores the dominant role of bacterial infections in driving MOF and mortality. Nonetheless, these findings call for a paradigm shift in the management of sAH, prioritising infection prevention and early targeted interventions as core principles of care in this high‐risk population.

## Author Contributions

M.B., J.S., B.M., and L.B. conceived the presented idea; L.B. performed the analysis. M.B. and L.B. wrote the manuscript, discussed the data, and generated figures and tables; all authors discussed the results and contributed to the final manuscript.

## Conflicts of Interest

J.S. served as the Chief Medical Officer of Vital Therapies Inc. at the time of the VTL‐308 study and is currently the Chief Medical Officer and Chairman of the Board of Albutec GmbH. H.W. served as a speaker/advisory board member for Abbott Laboratories & Abbott Molecular Inc., Bristol‐Myers Squibb, Gilead Sciences GmbH & Gilead Sciences Ltd., GlaxoSmithKline Services Unlimited, Janssen, Roche Diagnostics International Ltd., and Vir Biotechnology Inc.; received research support from Abbott Laboratories & Abbott Molecular Inc. and Biotest AG; and received lecture fees from Biotest AG and Gilead Sciences GmbH & Gilead Sciences Ltd. B.M. served as a speaker and/or advisory board member for AbbVIe, AstraZeneca, Falk, Fujirebio, Gilead, Luvos, MSD, Norgine, Roche, W. L. Gore & Associates and received research support from Altona Diagnostics, EWIMED, Fujirebio, and Roche, all unrelated to the present work.

## Supporting information


Figure S1.

**Table S1.** The table shows the inclusion and exclusion criteria of the VTL‐308 trial. AST, aspartate aminotransferase; MELD, model for end‐stage liver disease.
**Table S2.** Subgroup analyses of patients receiving ELAD therapy: Infection was a strong predictor of mortality (A), but the risk of infection development was not associated with corticosteroid intake (B). Subgroup analyses of patients receiving standard of care (SOC) therapy: Infections were linked to an elevated risk of death (C), but the risk of infections was not aggravated by corticosteroid use (D). CI, confidence interval; HR, hazard ratio; INR, international normalised ratio; MELD, model for end‐stage liver disease; WBC, white blood cell count.
**Table S3.** Predictors of infections. None of the variables analysed were associated with infection risk. The tables depict the respective hazard ratios (HR) and 95% confidence intervals (CI) from competing risk analyses for 90‐days of follow‐up. ALT, Alanine Aminotransferase; AP, alkaline phosphatase; AST, aspartate aminotransferase; ELAD, extracorporeal liver assist device; GAHS, Glasgow alcoholic hepatitis score; INR, International normalised ratio; MELD, model for end‐stage liver disease; WBC, white blood cells.
**Table S4.** Bacterial (A), but not fungal infections (B) were linked to an increased mortality in the competing risk and Cox regression analyses with time‐dependent covariates. CI, confidence interval; HR, hazard ratio; INR, International normalised ratio; WBC, white blood cell count.
**Table S5.** Bacterial infections were linked to an elevated likelihood of multi‐organ failure (MOF) (A). Fungal infections are not associated with a higher incidence of MOF (B). CI, confidence interval; HR, hazard ratio; INR, International normalised ratio.

## Data Availability

The data that support the findings of this study are available from the corresponding author upon reasonable request.

## References

[1] D. W. Crabb , G. Y. Im , G. Szabo , J. L. Mellinger , and M. R. Lucey , “Diagnosis and Treatment of Alcohol‐Associated Liver Diseases: 2019 Practice Guidance From the American Association for the Study of Liver Diseases,” Hepatology 71, no. 1 (2020): 306–333. 31314133 10.1002/hep.30866

[2] W. C. Maddrey , J. K. Boitnott , M. S. Bedine , F. L. Weber, Jr. , E. Mezey , and R. I. White, Jr. , “Corticosteroid Therapy of Alcoholic Hepatitis,” Gastroenterology 75, no. 2 (1978): 193–199. 352788

[3] M. R. Thursz , P. Richardson , M. Allison , et al., “Prednisolone or Pentoxifylline for Alcoholic Hepatitis,” New England Journal of Medicine 372, no. 17 (2015): 1619–1628. 25901427 10.1056/NEJMoa1412278

[4] E. Karakike , C. Moreno , and T. Gustot , “Infections in Severe Alcoholic Hepatitis,” Annals of Gastroenterology 30, no. 2 (2017): 152–160. 28243035 10.20524/aog.2016.0101 PMC5320027

[5] N. Vergis , S. R. Atkinson , S. Knapp , et al., “In Patients With Severe Alcoholic Hepatitis, Prednisolone Increases Susceptibility to Infection and Infection‐Related Mortality, and Is Associated With High Circulating Levels of Bacterial DNA,” Gastroenterology 152, no. 5 (2017): 1068–1077. 28043903 10.1053/j.gastro.2016.12.019 PMC6381387

[6] B. S. Hmoud , K. Patel , R. Bataller , and A. K. Singal , “Corticosteroids and Occurrence of and Mortality From Infections in Severe Alcoholic Hepatitis: A Meta‐Analysis of Randomized Trials,” Liver International 36, no. 5 (2016): 721–728. 26279269 10.1111/liv.12939

[7] N. Pyrsopoulos , T. Hassanein , R. Subramanian , et al., “A Study Investigating the Effect of Extracorporeal Cellular Therapy With C3A Cells on the Survival of Alcoholic Hepatitis Designed Along the Guidelines of the NIAAA,” Journal of Hepatology 70, no. 1 (2019): e282.

[8] L. Otero Sanchez , E. Karakike , H. Njimi , et al., “Clinical Course and Risk Factors for Infection in Severe Forms of Alcohol‐Associated Liver Disease,” Hepatology 74, no. 5 (2021): 2714–2724. 34046927 10.1002/hep.31984

[9] C. A. Arias and B. E. Murray , “The Rise of the Enterococcus: Beyond Vancomycin Resistance,” Nature Reviews. Microbiology 10, no. 4 (2012): 266–278. 22421879 10.1038/nrmicro2761 PMC3621121

[10] D. G. Maindad , S. Shenoy , S. Shenoy , S. Gopal , and B. V. Tantry , “Treatment of Hospital‐Acquired Infections in Patients With Cirrhosis—New Challenges,” Infection and Drug Resistance 15 (2022): 1039–1048. 35313728 10.2147/IDR.S283723 PMC8934116

[11] R. Jalan , J. Fernandez , R. Wiest , et al., “Bacterial Infections in Cirrhosis: A Position Statement Based on the EASL Special Conference 2013,” Journal of Hepatology 60, no. 6 (2014): 1310–1324. 24530646 10.1016/j.jhep.2014.01.024

[12] B. Kaur , R. Rosenblatt , and V. Sundaram , “Infections in Alcoholic Hepatitis,” Journal of Clinical and Translational Hepatology 10, no. 4 (2022): 718–725. 36062291 10.14218/JCTH.2022.00024 PMC9396323

[13] B. Leber , W. Spindelboeck , and V. Stadlbauer , “Infectious Complications of Acute and Chronic Liver Disease,” Seminars in Respiratory and Critical Care Medicine 33, no. 1 (2012): 80–95. 22447263 10.1055/s-0032-1301737

[14] A. M. Thulstrup , H. T. Sorensen , H. C. Schonheyder , J. K. Moller , and U. Tage‐Jensen , “Population‐Based Study of the Risk and Short‐Term Prognosis for Bacteremia in Patients With Liver Cirrhosis,” Clinical Infectious Diseases 31, no. 6 (2000): 1357–1361. 11096002 10.1086/317494

[15] M. Bartoletti , M. Giannella , R. E. Lewis , and P. Viale , “Bloodstream Infections in Patients With Liver Cirrhosis,” Virulence 7, no. 3 (2016): 309–319. 26864729 10.1080/21505594.2016.1141162 PMC4871664

[16] Y. Xie , B. Tu , Z. Xu , et al., “Bacterial Distributions and Prognosis of Bloodstream Infections in Patients With Liver Cirrhosis,” Scientific Reports 7, no. 1 (2017): 11482. 28904387 10.1038/s41598-017-11587-1 PMC5597589

[17] P. Boeira , H. Tan , E. Yates , and A. Dhanda , “Assessment of Immune Function and Prediction of Survival and Infection in Patients With Severe Alcoholic Hepatitis: An Exploratory Study,” JGH Open 7, no. 4 (2023): 286–290. 37125245 10.1002/jgh3.12891 PMC10134762

[18] A. Albillos , M. Lario , and M. Alvarez‐Mon , “Cirrhosis‐Associated Immune Dysfunction: Distinctive Features and Clinical Relevance,” Journal of Hepatology 61, no. 6 (2014): 1385–1396. 25135860 10.1016/j.jhep.2014.08.010

[19] J. D. Stange and B. Ibrahim , “OPAL but Not MARS Reverses Patient's Plasma Cytotoxicity in a Prospective Randomized Trial Using Extracorporeal Albumin Dialysis (ECAD),” Hepatology 76 (2022): 1244–1245.

[20] J. Leonhardt , R. S. Haider , C. Sponholz , et al., “Circulating Bile Acids in Liver Failure Activate TGR5 and Induce Monocyte Dysfunction,” Cellular and Molecular Gastroenterology and Hepatology 12, no. 1 (2021): 25–40. 33545429 10.1016/j.jcmgh.2021.01.011 PMC8082115

[21] J. Leonhardt , M. J. Dorresteijn , S. Neugebauer , et al., “Immunosuppressive Effects of Circulating Bile Acids in Human Endotoxemia and Septic Shock: Patients With Liver Failure Are at Risk,” Critical Care 27, no. 1 (2023): 372. 37759239 10.1186/s13054-023-04620-5 PMC10523742

[22] D. W. Cain and J. A. Cidlowski , “Immune Regulation by Glucocorticoids,” Nature Reviews. Immunology 17, no. 4 (2017): 233–247. 10.1038/nri.2017.1 PMC9761406 28192415

[23] O. Perez‐Hernandez , E. Gonzalez‐Reimers , A. Garcia‐Rodriguez , et al., “Value of Inflammatory Response and Oxidative Damage in the Diagnosis of Infections in Severe Alcoholic Hepatitis,” European Journal of Internal Medicine 119 (2024): 64–70. 37586986 10.1016/j.ejim.2023.08.005

[24] R. Moreau , R. Jalan , P. Gines , et al., “Acute‐On‐Chronic Liver Failure Is a Distinct Syndrome That Develops in Patients With Acute Decompensation of Cirrhosis,” Gastroenterology 144, no. 7 (2013): 1426–1437.e9. 23474284 10.1053/j.gastro.2013.02.042

[25] S. K. Sarin , A. Choudhury , M. K. Sharma , et al., “Acute‐On‐Chronic Liver Failure: Consensus Recommendations of the Asian Pacific Association for the Study of the Liver (APASL): An Update,” Hepatology International 13, no. 4 (2019): 353–390. 31172417 10.1007/s12072-019-09946-3 PMC6728300

[26] T. M. Lopes‐Secundo , T. Seva‐Pereira , B. R. Correa , et al., “Serum Sodium, Model for End‐Stage Liver Disease, and a Recent Invasive Procedure Are Risk Factors for Severe Acute‐On‐Chronic Liver Failure and Death in Cirrhotic Patients Hospitalized With Bacterial Infection,” European Journal of Gastroenterology & Hepatology 30, no. 9 (2018): 1055–1059. 29944488 10.1097/MEG.0000000000001184

[27] K. Gananandan , R. Singh , and G. Mehta , “Systematic Review and Meta‐Analysis of Biomarkers Predicting Decompensation in Patients With Compensated Cirrhosis,” BMJ Open Gastroenterology 11, no. 1 (2024): e001430. 10.1136/bmjgast-2024-001430 PMC11404266 39182920

[28] C. J. Wiedermann , “Hypoalbuminemia as Surrogate and Culprit of Infections,” International Journal of Molecular Sciences 22, no. 9 (2021): 4496. 33925831 10.3390/ijms22094496 PMC8123513

[29] C. R. Khatua , S. K. Sahu , D. Meher , et al., “Admission Serum Urea Is a Better Predictor of Mortality Than Creatinine in Patients With Acute‐On‐Chronic Liver Failure and Acute Kidney Injury,” Journal of Clinical and Experimental Hepatology 11, no. 5 (2021): 565–572. 34511817 10.1016/j.jceh.2020.12.005 PMC8414310

[30] J. Altamirano , C. Fagundes , M. Dominguez , et al., “Acute Kidney Injury Is an Early Predictor of Mortality for Patients With Alcoholic Hepatitis,” Clinical Gastroenterology and Hepatology 10, no. 1 (2012): 65–71.e3. 21946124 10.1016/j.cgh.2011.09.011

[31] A. Juanola , N. Tiwari , C. Sole , D. Adebayo , F. Wong , and P. Gines , “Organ Dysfunction and Failure in Liver Disease,” Liver International 45, no. 3 (2023): e15622. 37222263 10.1111/liv.15622

[32] R. Moreau , “Role of Infections in Acute‐On‐Chronic Liver Failure,” Digestive Diseases 33, no. 4 (2015): 577–581. 26159276 10.1159/000375356

[33] J. Thompson , N. Jones , A. Al‐Khafaji , et al., “Extracorporeal Cellular Therapy (ELAD) in Severe Alcoholic Hepatitis: A Multinational, Prospective, Controlled, Randomized Trial,” Liver Transplantation 24, no. 3 (2018): 380–393. 29171941 10.1002/lt.24986 PMC5873437

