# Supplementary material for: Bacterial Infections in Patients With Severe Alcohol‐Associated Hepatitis: Drivers of Organ Failure and Mortality
Source: Liver Int. 2025 May 7;45(6):e70111. doi: 10.1111/liv.70111 (PMC12057653; doi:10.1111/liv.70111)
Supplement: Supplementary file 1 — Figure S1. [file LIV-45-0-s001.pptx]

## Slide 1
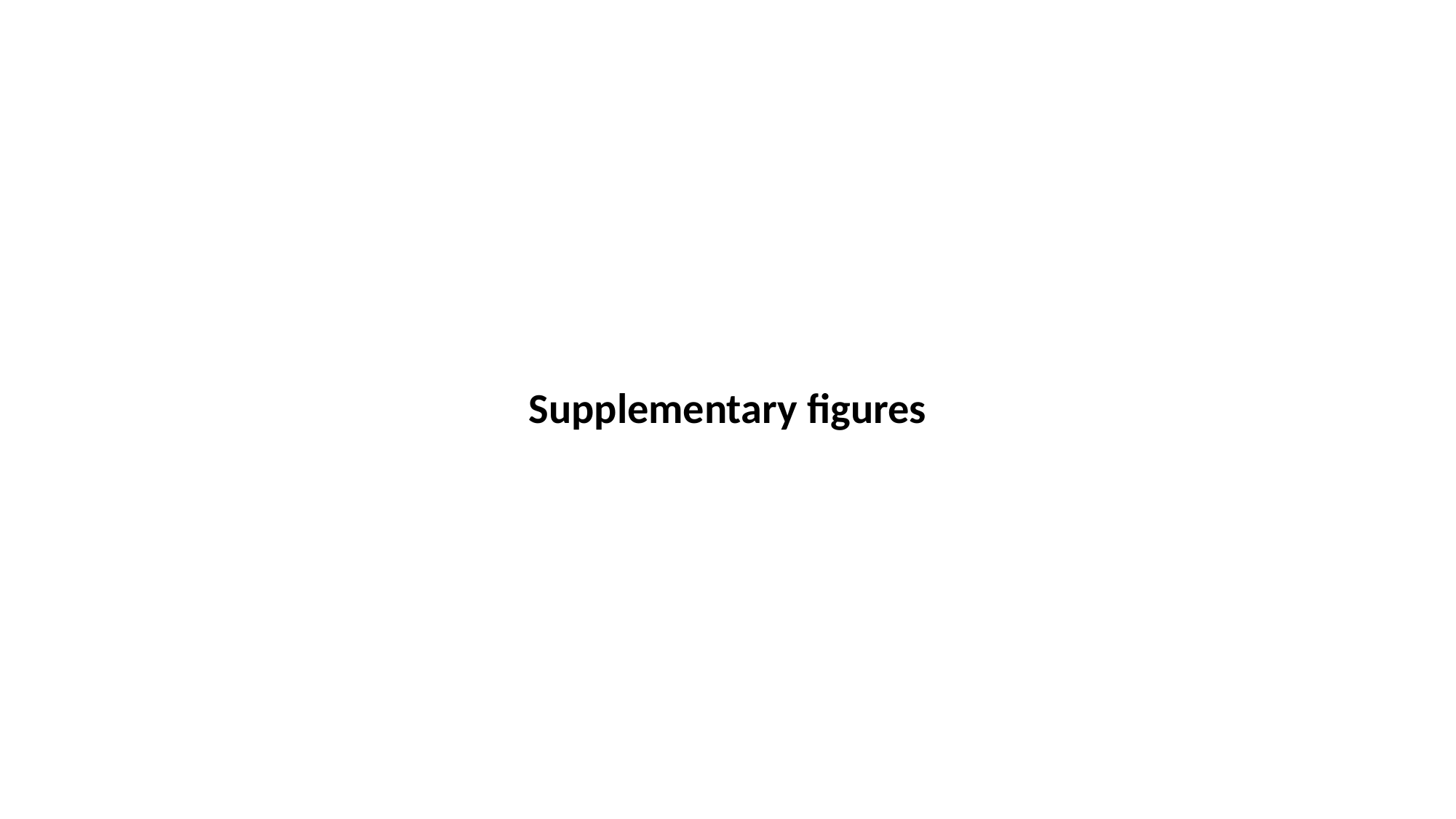

# Supplementary figures

## Slide 2
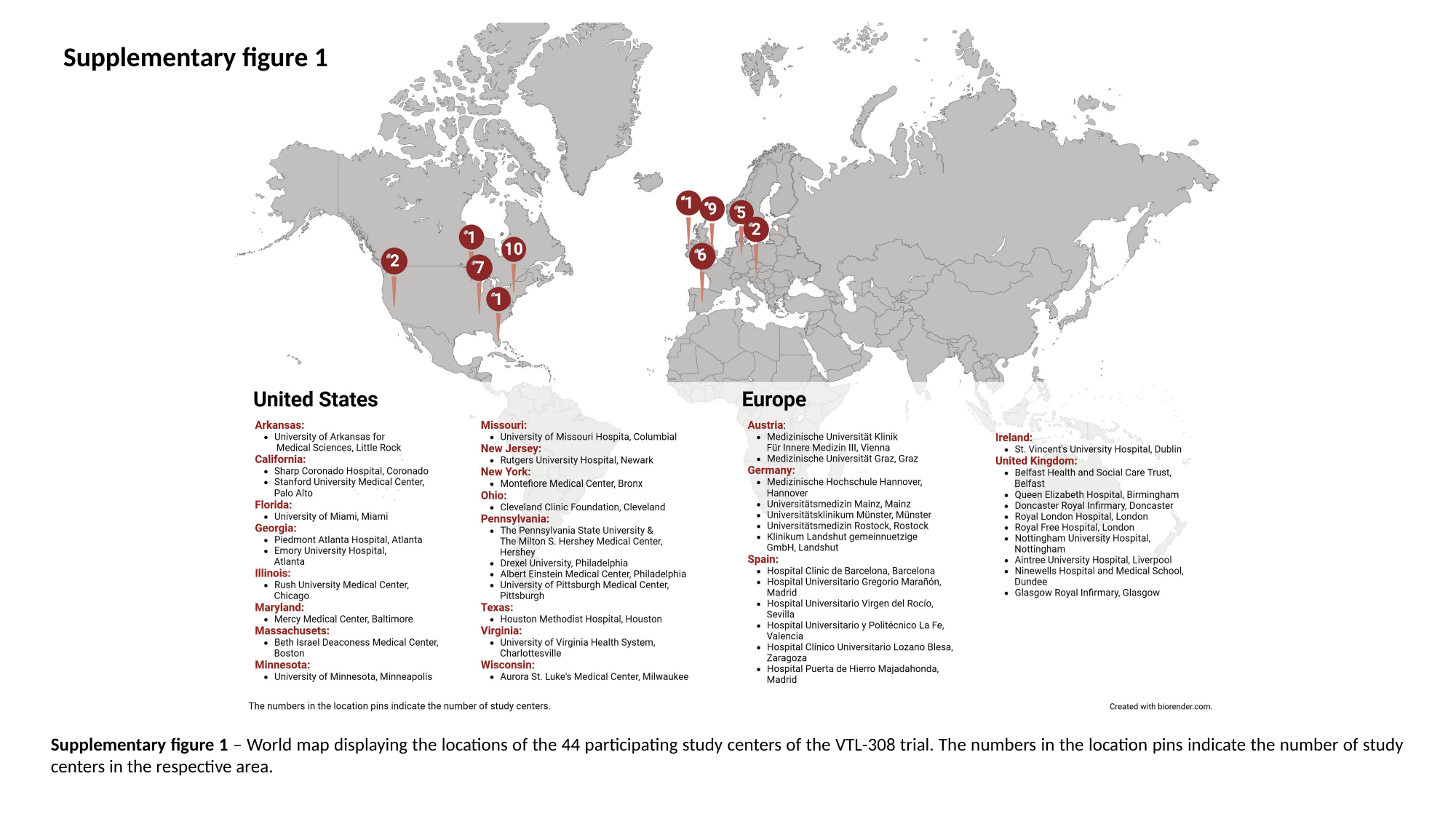

Supplementary figure 1
Supplementary figure 1 – World map displaying the locations of the 44 participating study centers of the VTL-308 trial. The numbers in the location pins indicate the number of study centers in the respective area.

## Slide 3
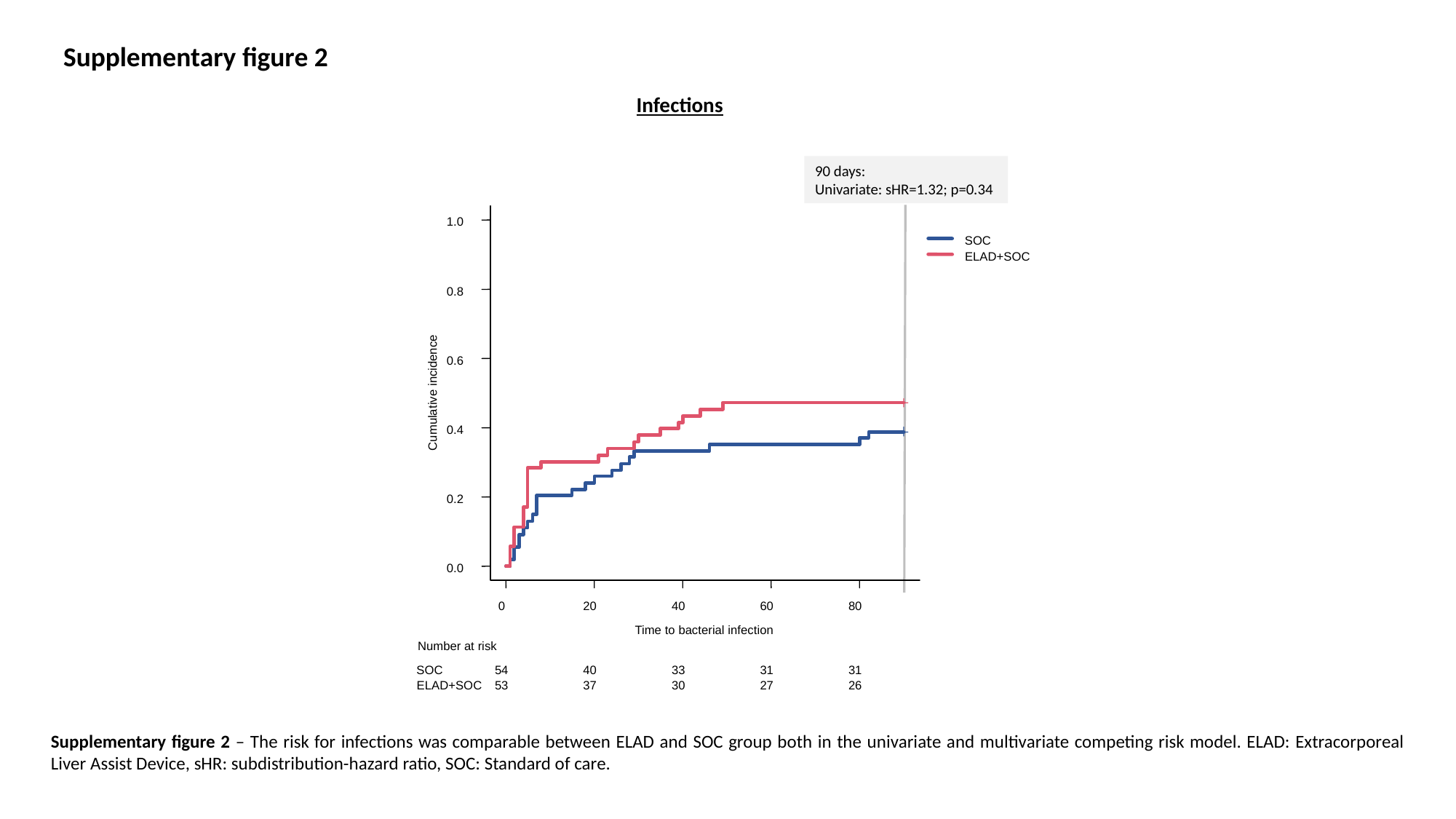

Supplementary figure 2
Infections
1.0
SOC
ELAD+SOC
0.8
0.6
Cumulative incidence
0.4
0.2
0.0
0
20
40
60
80
Time to bacterial infection
Number at risk
SOC
54
40
33
31
31
ELAD+SOC
53
37
30
27
26
90 days:
Univariate: sHR=1.32; p=0.34
Supplementary figure 2 – The risk for infections was comparable between ELAD and SOC group both in the univariate and multivariate competing risk model. ELAD: Extracorporeal Liver Assist Device, sHR: subdistribution-hazard ratio, SOC: Standard of care.

## Slide 4
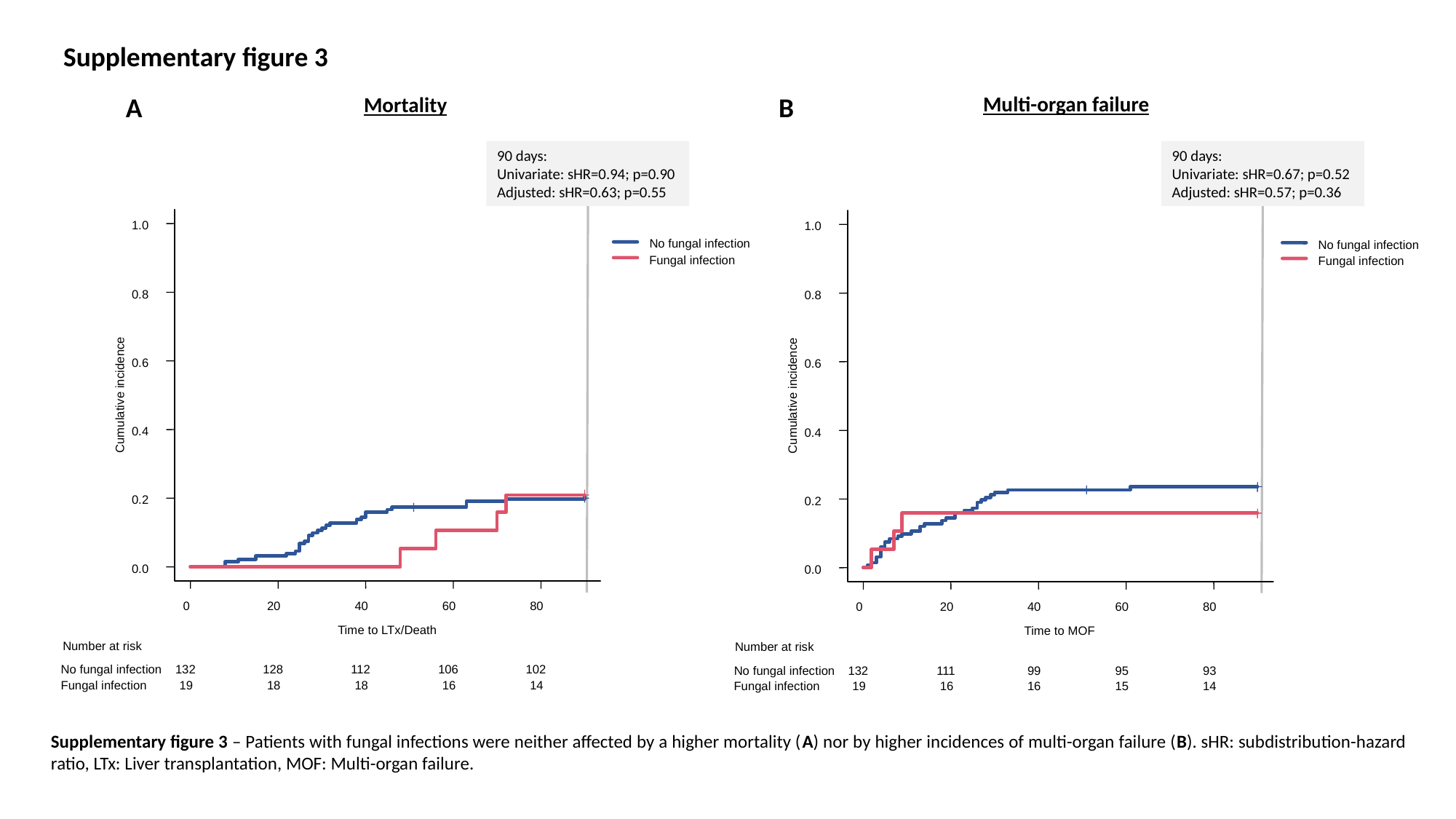

Supplementary figure 3
A
B
Multi-organ failure
Mortality
90 days:
Univariate: sHR=0.94; p=0.90
Adjusted: sHR=0.63; p=0.55
90 days:
Univariate: sHR=0.67; p=0.52
Adjusted: sHR=0.57; p=0.36
1.0
No fungal infection
Fungal infection
0.8
0.6
Cumulative incidence
0.4
0.2
0.0
0
20
40
60
80
Time to LTx/Death
Number at risk
No fungal infection
132
128
112
106
102
Fungal infection
19
18
18
16
14
1.0
No fungal infection
Fungal infection
0.8
0.6
Cumulative incidence
0.4
0.2
0.0
0
20
40
60
80
Time to MOF
Number at risk
No fungal infection
132
111
99
95
93
Fungal infection
19
16
16
15
14
Supplementary figure 3 – Patients with fungal infections were neither affected by a higher mortality (A) nor by higher incidences of multi-organ failure (B). sHR: subdistribution-hazard ratio, LTx: Liver transplantation, MOF: Multi-organ failure.
