# Supplementary material for: Bacterial Infections in Patients With Severe Alcohol‐Associated Hepatitis: Drivers of Organ Failure and Mortality
Source: Liver Int. 2025 May 7;45(6):e70111. doi: 10.1111/liv.70111 (PMC12057653; doi:10.1111/liv.70111)
Supplement: Supplementary file 2 — Table S1. The table shows the inclusion and exclusion criteria of the VTL‐308 trial. AST, aspartate aminotransferase; MELD, model for end‐stage liver disease. Table S2. Subgroup analyses of patients receiving ELAD therapy: Infection was a strong predictor of mortality (A), but the risk of infection development was not associated with corticosteroid intake (B). Subgroup analyses of patients receiving standard of care (SOC) therapy: Infections were linked to an elevated risk of death (C), but the risk of infections was not aggravated by corticosteroid use (D). CI, confidence interval; HR, hazard ratio; INR, international normalised ratio; MELD, model for end‐stage liver disease; WBC, white blood cell count. Table S3. Predictors of infections. None of the variables analysed were associated with infection risk. The tables depict the respective hazard ratios (HR) and 95% confidence intervals (CI) from competing risk analyses for 90‐days of follow‐up. ALT, Alanine Aminotransferase; AP, alkaline phosphatase; AST, aspartate aminotransferase; ELAD, extracorporeal liver assist device; GAHS, Glasgow alcoholic hepatitis score; INR, International normalised ratio; MELD, model for end‐stage liver disease; WBC, white blood cells. Table S4. Bacterial (A), but not fungal infections (B) were linked to an increased mortality in the competing risk and Cox regression analyses with time‐dependent covariates. CI, confidence interval; HR, hazard ratio; INR, International normalised ratio; WBC, white blood cell count. Table S5. Bacterial infections were linked to an elevated likelihood of multi‐organ failure (MOF) (A). Fungal infections are not associated with a higher incidence of MOF (B). CI, confidence interval; HR, hazard ratio; INR, International normalised ratio. [file LIV-45-0-s002.docx]

**Supplementary tables**

**Supplementary table 1**

| Inclusion | Exclusion |
| --- | --- |
| Age >=18 years | Age >=50 |
| Total bilirubin >=16 mg/dL (>=273.6 umol/L) | Platelet count <40,000/mm^3 |
| A clinical diagnosis of alcohol-induced liver decompensation (AILD) | International Normalized Ratio (INR) >2.5 |
| Maddrey score >=32 | Serum Creatinine >=1.3 mg/dL (>=115.04 umol/L) |
| Subjects must have severe acute alcoholic hepatitis (sAAH) diagnosed | MELD score >=30 |
| Subject or legally-authorized representative must provide Informed Consent | AST >500 IU/L |
| Subject must be eligible for Standard of Care treatment as defined in the protocol | Evidence of infection unresponsive to antibiotics |
|  | Evidence of reduction in total bilirubin of 20% or more in the previous 72 hours |
|  | Evidence of hemodynamic instability as defined specifically. |
|  | Evidence of active bleeding, major hemorrhage occurring within 48 hours prior to Randomization, or with banding of gastroesophageal varices during the 7 days immediately preceding screening |
|  | Clinical evidence of liver size reduction due to cirrhosis, unless Investigator interpretation otherwise and Sponsor agrees |
|  | Occlusive portal vein thrombosis impairing hepatopetal flow, or evidence of bile duct obstruction |
|  | Evidence by physical exam, history, or laboratory evaluation, of significant concomitant disease with a life expectancy of less than 3 months |
|  | Subject has chronic end-stage renal disease requiring chronic hemodialysis for more than 8 weeks (not classified as hepatorenal syndrome) |
|  | Subject ventilated or intubated |
|  | Subject on hemodialysis |
|  | Subject has liver disease related to homozygous hemachromotosis, Wilson's disease, has nonalcoholic fatty liver disease, or Budd-Chiari Syndrome |
|  | Serological evidence of active viral hepatitis A, B or C infection. If subject at risk for viral hepatitis A, B or C, and no serology is available, serologies must be obtained prior to Randomization |
|  | Pregnancy as determined by serum beta-human chorionic gonadotropin results, or subjects not willing to use effective means of contraception, without history of medical or surgical sterilization |
|  | Participation in another investigational drug, biologic, or device study within one month of enrollment, except for observational studies |
|  | Previous liver transplant |
|  | Previous enrollment in the treatment phase of another ELAD trial |
|  | Have a Do Not Resuscitate or a Do Not Intubate (DNR/DNI) directive or any other Advanced Directive limiting Standard of Care in place |
|  | Refusal to participate in the VTL-308E follow-up study |
|  | Inability to provide an address for home visits |

**Supplementary table 2A**

|  | Univariate | | | | Multivariable | | | |
| --- | --- | --- | --- | --- | --- | --- | --- | --- |
|  | **sHR** | **Lower 95% CI** | **Upper 95% CI** | **p value** | **HR** | **Lower 95% CI** | **Upper 95% CI** | **p value** |
| Competing risk analysis - 90 days of follow-up | | | | | | | | |
| Bacterial infection  Age  Bilirubin  INR  WBC  Urea  Liver cirrhosis | 8.60 | 1.13 | 65.18 | 0.037 | 15.58  1.12  1.08  1.40  1.01  1.02  3.92 | 3.38  0.99  1.01  0.23  0.95  0.97  0.59 | 71.78  1.26  1.15  8.48  1.08  1.07  25.92 | <0.001  0.076  0.018  0.710  0.690  0.520  0.160 |
| Time dependent covariate - 90 days of follow-up | | | | | | | | |
|  | **HR** | **Lower 95% CI** | **Upper 95% CI** | **p value** | **HR** | **Lower 95% CI** | **Upper 95% CI** | **p value** |
| Bacterial infection  Age  Bilirubin  INR  Leukocytes  Urea  Liver cirrhosis | 5.995 | 1.36 | 26.42 | 0.01796 | 24.47  1.11  1.19  2.27  1.01  1.00  4.65 | 3.89  1.01  1.08  0.36  0.93  0.95  0.76 | 153.90  1.22  1.30  14.30  1.09  1.06  28.34 | <0.001  0.039  <0.001  0.38  0.854  0.930  0.096 |

**Supplementary table 2B**

|  | Univariate | | | | Multivariable | | | |
| --- | --- | --- | --- | --- | --- | --- | --- | --- |
|  | **sHR** | **Lower 95% CI** | **Upper 95% CI** | **p value** | **HR** | **Lower 95% CI** | **Upper 95% CI** | **p value** |
| 90 days | | | | | | | | |
| Steroids | 0.65 | 0.30 | 1.43 | 0.290 | 0.65 | 0.28 | 1.50 | 0.310 |
| MELD | 0.96 | 0.82 | 1.12 | 0.570 | 1.02 | 0.84 | 1.23 | 0.850 |
| Previous infection (30 days) | 0.39 | 0.10 | 1.49 | 0.170 | 0.36 | 0.10 | 1.22 | 0.100 |
| Liver cirrhosis | 0.57 | 2.67 | 1.21 | 0.140 | 0.47 | 0.19 | 1.14 | 0.095 |
| Time-censored corticosteroid use – 90 days | | | | | | | | |
|  | **HR** | **Lower 95% CI** | **Upper 95% CI** | **p value** | **HR** | **Lower 95% CI** | **Upper 95% CI** | **p value** |
| Steroids | 0.47 | 0.18 | 1.26 | 0.130 | 0.44 | 0.14 | 1.32 | 0.140 |
| MELD | 0.88 | 0.74 | 1.05 | 0.160 | 0.97 | 0.77 | 1.21 | 0.770 |
| Previous infection (30 days) | 0.40 | 0.11 | 1.42 | 0.160 | 0.32 | 0.12 | 0.86 | 0.025 |
| Liver cirrhosis | 0.48 | 0.21 | 1.12 | 0.088 | 0.37 | 0.11 | 1.18 | 0.093 |

**Supplementary table 2C**

|  | Univariate | | | | Multivariable | | | |
| --- | --- | --- | --- | --- | --- | --- | --- | --- |
|  | **sHR** | **Lower 95% CI** | **Upper 95% CI** | **p value** | **HR** | **Lower 95% CI** | **Upper 95% CI** | **p value** |
| Competing risk analysis - 90 days of follow-up | | | | | | | | |
| Bacterial infection  Age  Bilirubin  INR  WBC  Urea  Liver cirrhosis | 4.20 | 1.22 | 14.52 | 0.023 | 8.42  1.08  1.08  6.72  0.97  1.03  0.60 | 1.44  0.99  1.00  1.58  0.92  1.01  0.17 | 49.46  1.18  1.18  28.55  1.02  1.05  2.07 | 0.02  0.098  0.061  0.010  0.200  0.001  0.420 |
| Time dependent covariate - 90 days of follow-up | | | | | | | | |
|  | **HR** | **Lower 95% CI** | **Upper 95% CI** | **p value** | **HR** | **Lower 95% CI** | **Upper 95% CI** | **p value** |
| Bacterial infection  Age  Bilirubin  INR  WBC  Urea  Liver cirrhosis | 4.33 | 1.44 | 13.07 | 0.009 | 4.68  1.08  1.06  6.08  0.97  1.05  0.75 | 1.45  0.99  0.97  1.21  0.93  1.02  0.22 | 15.11  1.17  1.15  30.62  1.02  1.07  2.56 | 0.010  0.072  0.194  0.029  0.213  <0.001  0.646 |

**Supplementary table 2D**

|  | Univariate | | | | Multivariable | | | |
| --- | --- | --- | --- | --- | --- | --- | --- | --- |
|  | **sHR** | **Lower 95% CI** | **Upper 95% CI** | **p value** | **HR** | **Lower 95% CI** | **Upper 95% CI** | **p value** |
| 90 days | | | | | | | | |
| Steroids | 0.89 | 0.38 | 2.06 | 0.780 | 1.07 | 0.44 | 2.59 | 0.890 |
| MELD | 1.16 | 0.95 | 1.43 | 0.150 | 1.16 | 0.94 | 1.43 | 0.160 |
| Previous infection (30 days) | 1.74 | 0.67 | 4.51 | 0.250 | 1.49 | 0.57 | 3.89 | 0.420 |
| Liver cirrhosis | 1.68 | 0.61 | 4.65 | 0.320 | 1.58 | 0.56 | 4.47 | 0.390 |
| Time-censored corticosteroid use – 90 days | | | | | | | | |
|  | **HR** | **Lower 95% CI** | **Upper 95% CI** | **p value** | **HR** | **Lower 95% CI** | **Upper 95% CI** | **p value** |
| Steroids | 0.50 | 0.16 | 1.55 | 0.230 | 0.64 | 0.19 | 2.13 | 0.470 |
| MELD | 1.34 | 0.95 | 1.87 | 0.093 | 1.24 | 0.90 | 1.70 | 0.190 |
| Previous infection (30 days) | 1.48 | 0.51 | 4.32 | 0.470 | 1.21 | 0.40 | 3.70 | 0.740 |
| Liver cirrhosis | 1.95 | 0.51 | 7.36 | 0.330 | 1.57 | 0.48 | 5.17 | 0.460 |

**Supplementary table 3**

|  | 90 days | | | |
| --- | --- | --- | --- | --- |
|  | **sHR** | **Lower 95% CI** | **Upper 95% CI** | **p value** |
| Age | 1.01 | 0.97 | 1.05 | 0.710 |
| WBC | 0.98 | 0.94 | 1.03 | 0.410 |
| Urea | 0.99 | 0.97 | 1.01 | 0.360 |
| INR | 1.28 | 0.52 | 3.15 | 0.590 |
| Creatinine | 0.50 | 0.13 | 1.89 | 0.310 |
| Bilirubin | 1.00 | 0.96 | 1.05 | 0.930 |
|  |  |  |  |  |
| Maddrey | 1.01 | 0.99 | 1.02 | 0.570 |
| GAHS | 0.84 | 0.58 | 1.23 | 0.380 |
| MELD | 1.03 | 0.90 | 1.17 | 0.690 |
| De ritis ratio | 1.04 | 0.97 | 1.12 | 0.250 |
|  |  |  |  |  |
| Steroids (BL) | 0.74 | 0.42 | 1.33 | 0.320 |
| ELAD (PP) | 1.32 | 0.75 | 2.32 | 0.340 |
| Diabetes | 1.26 | 0.55 | 2.91 | 0.590 |
| Previous infection (30 days) | 0.88 | 0.41 | 1.86 | 0.730 |
| Hemoglobin | 1.06 | 0.92 | 1.23 | 0.430 |
| AST | 1.00 | 1.00 | 1.01 | 0.080 |
| ALT | 1.01 | 1.00 | 1.01 | 0.200 |
| Albumin | 1.10 | 0.68 | 1.78 | 0.700 |
| Lactic acid | 0.80 | 0.48 | 1.35 | 0.400 |
| Body temperature | 1.21 | 0.76 | 1.92 | 0.420 |
|  |  |  |  |  |
| Length of hospital stay before study inclusion | 1.00 | 0.97 | 1.04 | 0.790 |
| Ascites (BL) | 1.01 | 0.41 | 2.50 | 0.980 |
| Moderate/severe ascites (BL) | 1.48 | 0.84 | 2.62 | 0.170 |
| Liver cirrhosis | 0.93 | 0.51 | 1.71 | 0.820 |
| Malnutrition | 1.52 | 0.83 | 2.77 | 0.180 |
| Hepatic encephalopathy (BL) | 1.26 | 0.71 | 2.23 | 0.430 |

**Supplementary table 4A**

|  | Univariate | | | | Multivariable | | | |
| --- | --- | --- | --- | --- | --- | --- | --- | --- |
|  | **sHR** | **Lower 95% CI** | **Upper 95% CI** | **p value** | **HR** | **Lower 95% CI** | **Upper 95% CI** | **p value** |
| Competing risk analysis - 90 days of follow-up | | | | | | | | |
| Bacterial infection  Age  Bilirubin  INR  WBC  Urea  Liver cirrhosis | 5.22 | 1.83 | 14.90 | 0.002 | 7.78  1.10  1.08  3.65  0.98  1.02  1.12 | 2.44  1.02  1.03  1.19  0.95  1.01  0.41 | 24.86  1.17  1.13  11.14  1.02  1.04  3.05 | <0.001  0.010  <0.001  0.023  0.390  0.012  0.830 |
| Time dependent covariate - 90 days of follow-up | | | | | | | | |
|  | **HR** | **Lower 95% CI** | **Upper 95% CI** | **p value** | **HR** | **Lower 95% CI** | **Upper 95% CI** | **p value** |
| Bacterial infection  Age  Bilirubin  INR  WBC  Urea  Liver cirrhosis | 4.80 | 1.99 | 11.57 | <0.001 | 7.19  1.08  1.10  3.52  0.98  1.03  1.46 | 2.83  1.02  1.05  1.14  0.94  1.02  0.57 | 18.25  1.15  1.16  10.83  1.02  1.05  3.77 | <0.001  0.008  <0.001  0.028  0.250  <0.001  0.431 |

**Supplementary table 4B**

|  | Univariate | | | | Multivariable | | | |
| --- | --- | --- | --- | --- | --- | --- | --- | --- |
|  | **sHR** | **Lower 95% CI** | **Upper 95% CI** | **p value** | **HR** | **Lower 95% CI** | **Upper 95% CI** | **p value** |
| Competing risk analysis - 90 days of follow-up | | | | | | | | |
| Fungal infection  Age  Bilirubin  INR  WBB  Urea  Liver cirrhosis | 0.94 | 0.36 | 2.49 | 0.900 | 0.63  1.09  1.07  3.85  1.00  1.01  1.26 | 0.14  1.02  1.02  1.35  0.95  0.99  0.45 | 2.87  1.18  1.12  10.98  1.04  1.03  3.57 | 0.550  0.019  0.003  0.012  0.850  0.230  0.660 |
| Time dependent covariate - 90 days of follow-up | | | | | | | | |
|  | **HR** | **Lower 95% CI** | **Upper 95% CI** | **p value** | **HR** | **Lower 95% CI** | **Upper 95% CI** | **p value** |
| Fungal infection  Age  Bilirubin  INR  WBB  Urea | 1.46 | 0.56 | 3.77 | 0.439 | 1.36  1.07  1.08  4.38  0.99  1.03 | 0.49  1.01  1.03  1.45  0.95  1.01 | 3.76  1.14  1.13  13.22  1.03  1.04 | 0.550  0.020  0.003  0.009  0.514  0.003 |

**Supplementary table 5A**

|  | Univariate | | | | Multivariable | | | |
| --- | --- | --- | --- | --- | --- | --- | --- | --- |
|  | **sHR** | **Lower 95% CI** | **Upper 95% CI** | **p value** | **HR** | **Lower 95% CI** | **Upper 95% CI** | **p value** |
| Competing risk analysis - 90 days of follow-up | | | | | | | | |
| Bacterial infection  Age  Bilirubin  INR  WBB  Urea  Liver cirrhosis | 2.31 | 1.03 | 5.17 | 0.042 | 2.46  1.06  1.05  1.68  0.99  1.02  2.11 | 1.12  1.00  1.01  0.62  0.96  1.00  0.86 | 5.39  1.12  1.10  4.52  1.03  1.04  5.17 | 0.025  0.039  0.015  0.310  0.650  0.015  0.100 |
| Time dependent covariate - 90 days of follow-up | | | | | | | | |
|  | **HR** | **Lower 95% CI** | **Upper 95% CI** | **p value** | **HR** | **Lower 95% CI** | **Upper 95% CI** | **p value** |
| Bacterial infection  Age  Bilirubin  INR  WBB  Urea  Liver cirrhosis | 1.99 | 1.00 | 3.96 | 0.050 | 2.46  1.07  1.06  1.71  0.99  1.02  2.12 | 1.18  1.01  1.01  0.59  0.95  1.00  0.82 | 5.11  1.13  1.12  4.93  1.03  1.04  5.45 | 0.016  0.027  0.023  0.324  0.590  0.031  0.120 |

**Supplementary table 5B**

|  | Univariate | | | | Multivariable | | | |
| --- | --- | --- | --- | --- | --- | --- | --- | --- |
|  | **sHR** | **Lower 95% CI** | **Upper 95% CI** | **p value** | **HR** | **Lower 95% CI** | **Upper 95% CI** | **p value** |
| Competing risk analysis - 90 days of follow-up | | | | | | | | |
| Fungal infection  Age  Bilirubin  INR  Leukocytes  Urea  Liver cirrhosis | 0.67 | 0.20 | 2.28 | 0.520 | 0.57  1.07  1.05  2.64  1.00  1.02  1.97 | 0.17  1.01  1.01  1.85  0.96  1.00  0.81 | 1.88  1.14  1.10  5.91  1.04  1.03  4.78 | 0.360  0.027  0.015  0.100  1.000  0.037  0.140 |
| Time dependent covariate - 90 days of follow-up | | | | | | | | |
|  | **HR** | **Lower 95% CI** | **Upper 95% CI** | **p value** | **HR** | **Lower 95% CI** | **Upper 95% CI** | **p value** |
| Fungal infection  Age  Bilirubin  INR  Leukocytes  Urea | 0.43 | 0.06 | 3.17 | 0.41 | 0.44  1.07  1.05  2.23  1.00  1.01 | 0.06  1.01  1.00  0.78  0.96  1.00 | 3.34  1.14  1.11  6.35  1.04  1.03 | 0.426  0.022  0.039  0.135  1.000  0.090 |
